# Morphology–Coordination Coupling of Fe–TCPP and g-C_3_N_4_ Nanotubes for Enhanced ROS Generation and Visible-Light Photocatalysis

**DOI:** 10.3390/nano15191465

**Published:** 2025-09-24

**Authors:** Nannan Zheng, Yulan Zhang, Chunlei Dong, Zhiming Chen, Jianbin Chen

**Affiliations:** 1 Anhui Engineering Research Center of Highly Reactive Micro-Nano Powders, Chizhou University, Chizhou 247000, China; zhengnan@czu.edu.cn (N.Z.);; 2 School of Applied Physics and Materials, Wuyi University, Jiangmen 529020, China

**Keywords:** graphitic carbon nitride nanotubes, Fe–TCPP, Fe–N coordination, reactive oxygen species, photocatalysis, visible light

## Abstract

Fe–porphyrin/g-C_3_N_4_ composites have emerged as promising visible-light photocatalysts, but their performance remains limited by inefficient charge separation and low reactive oxygen species (ROS) yield. Here, iron–tetra(4-carboxyphenyl) porphyrin (Fe–TCPP) was coupled with g-C_3_N_4_ nanotubes (CNNTs) via a facile self-assembly strategy, creating a morphology-coordinated system. Comprehensive characterization (XRD, FTIR, SEM/TEM, BET, UV–Vis diffuse reflectance, PL, XPS, and EPR) confirmed the structural integrity, electronic coupling, and ROS generation capability of the composites. Fe–TCPP incorporation narrowed the bandgap from 2.78 to 2.56 eV, prolonged the average carrier lifetime from 6.3 to 7.5 ns, and significantly enhanced the generation of •OH and ^1^O_2_. The optimized 1 wt% Fe–TCPP@CNNTs achieved complete Rhodamine B degradation within 30 min under visible light, with the highest two-stage apparent rate constants (k_1_ = 0.0964 min^−1^, k_2_ = 0.328 min^−1^). In addition, the hybrids retained over 90% activity after six consecutive runs, confirming their stability and recyclability. The synergistic effect of Fe–N coordination and nanotubular architecture thus promotes light harvesting, charge separation, and ROS utilization, offering a promising design principle for high-performance photocatalysts in environmental remediation.

## 1. Introduction

Water pollution caused by dye-containing wastewater has become a major environmental concern worldwide because many dyes are chemically stable, toxic, and resistant to conventional treatments [1]. Photocatalysis, which harnesses solar or artificial light to drive oxidative degradation, is a promising strategy for water purification owing to its environmental compatibility and ability to mineralize organics without generating secondary pollution [2]. Among various photocatalysts, graphitic carbon nitride (g-C_3_N_4_) has attracted considerable interest due to its suitable bandgap (~2.7 eV), high thermal stability, and facile synthesis from inexpensive precursors [3,4,5,6,7,8]. However, pristine g-C_3_N_4_ suffers from rapid electron–hole recombination, a relatively small surface area, and limited visible-light absorption, which collectively restrict its photocatalytic activity [3,9,10,11,12]. Morphology engineering has proven effective for improving g-C_3_N_4_ performance. Nanosheets increase the number of exposed active sites [13,14,15,16,17], whereas nanotubes facilitate directional charge transport, suppress recombination, and enhance light harvesting via multiple-scattering effects [18,19,20,21]. In parallel, porphyrin-based compounds are widely used as photosensitizers and catalytic centers because of their strong visible-light absorption, tunable electronic structures, and metal-coordination capability [8,16,22,23,24,25,26,27,28]. Incorporating iron-tetra(4-carboxyphenyl) porphyrin (Fe–TCPP) into g-C_3_N_4_ can extend the absorption range and introduce Fe–N coordination sites that promote charge separation and activate molecular oxygen to generate reactive oxygen species (ROS) [29]. Despite these advantages, most reported Fe-porphyrin/g-C_3_N_4_ systems rely on nanosheets and seldom interrogate how morphology control and coordination chemistry act synergistically, nor do they provide direct evidence of dual ROS pathways (•OH and ^1^O_2_).

Here, we develop a morphology-coordinated photocatalyst by integrating Fe–TCPP with g-C_3_N_4_ nanotubes (CNNTs) via a simple self-assembly method. The combination of Fe–N coordination and nanotubular architecture works synergistically to broaden light absorption, enhance charge separation, and boost ROS formation. Mechanistic experiments coupling electron paramagnetic resonance (EPR) with radical-quenching tests provide direct evidence for the coexistence of •OH and ^1^O_2_ pathways, while time-resolved photoluminescence (TRPL) indicates prolonged carrier lifetimes. The optimized 1 wt% Fe–TCPP@CNNTs achieve complete RhB degradation within 30 min under visible light and demonstrate stable recyclability across repeated runs, underscoring their potential for practical environmental applications.

In addition to morphology and coordination effects, structural factors such as dimensional confinement, intrinsic defects, and the nanotube diameter can strongly influence the photocatalytic process, and these aspects are discussed to contextualize quantum-size effects and stability. Moreover, theoretical modeling approaches (e.g., density functional theory and molecular dynamics) offer complementary insights into charge–transfer dynamics, while the structural and electronic features of Fe–TCPP@CNNTs also suggest promise in solar fuel production and visible-light-driven organic synthesis.

## 2. Materials and Methods

### 2.1. Chemicals and Reagents

All chemicals and solvents were of analytical grade and used without further purification. Urea, melamine, methanol, and FeCl_3_ were obtained from Aladdin Reagent (Shanghai, China). 5,10,15,20-Tetrakis(4-carboxyphenyl) porphyrin (H_2_TCPP) was purchased from Zhengzhou Acme Chemicals Co., Ltd. (Zhengzhou, China).

### 2.2. Preparation of Fe–TCPP

Iron–tetra(4-carboxyphenyl) porphyrin (Fe–TCPP) was synthesized following reported procedures [30,31]. Briefly, solution A was prepared by dissolving 20 mg of H_2_TCPP in 10 mL of DMF/n-hexane (*v*/*v* = 1:3). Solution B was prepared by dissolving 10 mg of FeCl_3_ in 10 mL of ethanol. Solution B was added dropwise to solution A under stirring, and the mixture was heated at 80 °C for 12 h. The resulting brown precipitate was collected by centrifugation, washed three times with ethanol, and oven-dried to give Fe–TCPP as a brown powder.

### 2.3. Preparation of g-C_3_N_4_ Nanosheets and Nanotubes

g-C_3_N_4_ nanomaterials were synthesized by thermal polymerization in air. Urea and melamine were mixed in a defined ratio, ground, and placed in a covered crucible. The crucible was heated in a muffle furnace from room temperature to 550 °C at 5 °C/min under an air atmosphere, held for 4 h, and then naturally cooled. The pale-yellow product was washed with deionized water and ethanol and dried at 80 °C. A urea/melamine ratio of 4:1 yielded nanosheets (CNNSs), while a ratio of 9:1 produced nanotubes (CNNTs), consistent with nucleation and growth pathways governed by precursor composition.

### 2.4. Preparation of Fe–TCPP@g-C_3_N_4_

**Scheme 1:**
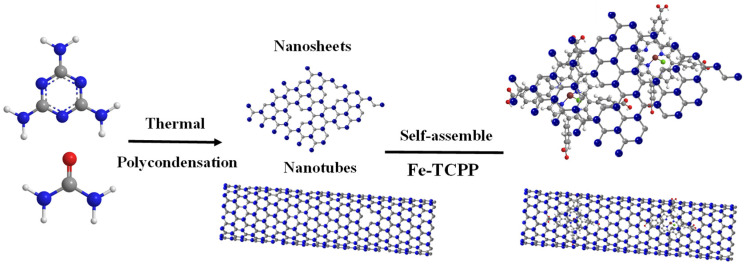
Synthesis diagram of Fe–TCPP@g-C_3_N_4_ nanomaterials.

Fe–TCPP@g-C_3_N_4_ composites were synthesized via a self-assembly method (Scheme 1) [24]. Typically, Fe–TCPP and g-C_3_N_4_ were weighed in a 1:100 mass ratio and dispersed in 50 mL of ethanol. The suspension was stirred at 50 °C for 5 h, centrifuged at 8000 rpm for 10 min, washed twice with ethanol, and dried at 60 °C under a vacuum. A series of composites with 1, 5, 10, and 20 wt% Fe–TCPP loading were prepared under identical conditions.

### 2.5. Characterization

The crystal structure was analyzed by X-ray diffraction (XRD, Dandong Haoyuan) using Cu Kα radiation (λ = 1.54187 Å) over 2θ = 10–50° at 0.02° s^−1^. Fourier transform infrared (FT-IR) spectra were recorded on a Thermo Fisher Nicolet iS10 spectrometer in the 400–4000 cm^−1^ range. Morphology and elemental mapping were examined by field-emission scanning electron microscopy (FE-SEM, 15 kV) with an EDS detector. UV–Vis diffuse reflectance spectra (DRS) were measured on a Hitachi U-9000 spectrophotometer with an integrating sphere (200–700 nm). X-ray photoelectron spectroscopy (XPS, Thermo K-Alpha) was performed with Al Kα radiation (hν = 1486.68 eV) under 5 × 10^−10^ Pa. Steady-state photoluminescence (PL) spectra were recorded on a Shimadzu RF-5301PC spectrophotometer at 310 nm excitation. N_2_ adsorption–desorption isotherms were measured at 77 K on a Micromeritics ASAP 2020 analyzer. Electron paramagnetic resonance (EPR, Bruker EMXplus) spectra were collected at the X-band (~9.4 GHz) and room temperature. DMPO, TEMP, and BQ were employed as spin traps for •OH, ^1^O_2_, and O_2_•^−^, respectively, at concentrations of XX mM under visible-light irradiation (λ > 420 nm, 300 W Xe lamp).

### 2.6. Measurement of Photocatalytic Activity

The photocatalytic activity of the synthesized materials was evaluated by monitoring the degradation of Rhodamine B (RhB) under visible-light irradiation in a PCX-50C Discover Multichannel Photochemical Reaction System (Beijing Procelight), which comprises five quartz reactors each equipped with a white-LED lamp (light intensity 500 mW cm^−2^). In a typical run, 20 mg of catalyst was dispersed in 50 mL of 10 mg L^−1^ RhB solution and magnetically stirred in the dark for 30 min to establish adsorption–desorption equilibrium. The suspension was then irradiated, and 3 mL aliquots were withdrawn every 10 min, filtered through a 0.22 µm membrane, and analyzed by UV–Vis spectrophotometry at 554 nm to determine the RhB concentration. The degradation efficiency (*R_t_*) was calculated according to Equation (1):

(1) $$R_{t}=\left(1-\frac{C_{t}}{C_{o}}\right)\times 100\%$$

where $R_{t}\;\;$ represents the degradation efficiency (%), $C_{o}$ (mg/L) is the initial RhB concentration, and $C_{t}$ (mg/L) is the RhB concentration at a given reaction time t. This equation provides a quantitative measure of the photocatalytic performance by comparing the RhB concentration at different time intervals to its initial value.

## 3. Results and Discussion

### 3.1. Structural and Morphological Characterization

**Figure 1:**
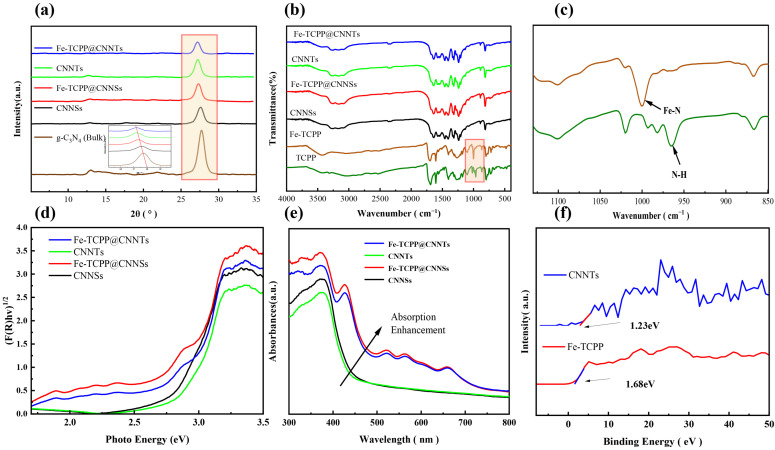
(**a**) XRD patterns; (**b**) FTIR spectra; (**c**) enlarged view of FTIR spectra highlighting characteristic peaks of Fe–TCPP and TCPP; (**d**) Tauc plots derived from UV–Vis DRS spectra; (**e**) corresponding absorption spectra; (**f**) XPS valence band spectra.

X-ray diffraction (XRD) analysis (Figure 1a) showed that both pristine g-C_3_N_4_ and Fe–TCPP@g-C_3_N_4_ retain the two characteristic reflections at ~13° and ~27°, assigned to the (100) in-plane ordering of tri-s-triazine rings and the (002) interlayer π-stacking of graphitic sheets, respectively [32,33]. Compared with bulk, the nanosheet and nanotube morphologies exhibit progressively weakened and broadened (100) and (002) peaks, reflecting reduced crystallite dimensions and layer stacking caused by NH_3_ release during urea decomposition. Specifically, the (002) peak shifts from 27.7° (bulk) to 27.6° (nanosheets) and 27.3° (nanotubes), consistent with increased disorder and exfoliation-induced curvature. Upon Fe–TCPP incorporation, the (002) reflection moves further to 27.4° in Fe–TCPP@CNNSs and 27.2° in Fe–TCPP@CNNTs, implying an interlayer expansion of 0.02–0.02 Å via Bragg’s law. This slight expansion is too small to represent true intercalation of the bulky porphyrin macrocycle; instead, it is more plausibly attributed to surface π–π stacking, edge coordination, and defect-induced microstrain, in agreement with recent modeling studies [34,35]. The absence of distinct Fe–TCPP peaks confirms its amorphous dispersion within the g-C_3_N_4_ matrix, where it likely intercalates between layers through coordination or π–π interactions [36,37,38].

FT-IR spectra (Figure 1b,c) provide complementary insights into key functional groups. In free TCPP, the N–H out-of-plane deformation stretching vibration of the pyrrole ring appears at ~966 cm^−1^. Upon Fe–TCPP, this feature disappears and a new band emerges at ~1000 cm^−1^, attributable to Fe–N stretching and confirming successful incorporation of iron into the porphyrin core [39]. Pristine g-C_3_N_4_ exhibits characteristic absorptions at 811 cm^−1^ (out-of-plane bending of tri-s-triazine rings), 1238, 1315, and 1405 cm^−1^ (aromatic C–N stretching of heptazine units), and 1635 cm^−1^ (C=N stretching of the conjugated tri-s-triazine framework) [40]. These remain unchanged in the composites, indicating preservation of the heptazine backbone. The Fe–N band (~1000 cm^−1^) and other porphyrin modes in the 1200–1600 cm^−1^ region are not clearly resolved in the composites due to low loading and spectral overlap with intense g-C_3_N_4_ bands, but their contribution is indirectly supported by XPS analysis.

UV–Vis diffuse reflectance and absorbance spectra (Figure 1d,e) were transformed using the Kubelka–Munk function and fitted via Tauc relations to estimate optical band gaps (details in Figure S1). Treating CNNSs, Fe–TCPP@CNNSs, CNNTs and Fe–TCPP@CNNTs as indirect semiconductors and Fe–TCPP as a direct semiconductor yields $E_{g}$ values of 2.75, 2.54, 2.78, 2.56 and 1.98 eV, respectively. CNNTs show a slightly larger bandgap but a blue-shifted edge relative to CNNSs, which is rationalized not only by tubular versus flat geometry but also by their two-dimensional curved nature that introduces additional confinement. This curvature modifies the spatial distribution of electronic states, enhances charge localization, and amplifies quantum-size effects compared with flat nanosheets. Their stronger apparent absorption between 500 and 800 nm arises from enhanced light–matter interaction due to tubular geometry and multiple scattering, which lengthen the optical path despite the marginally wider gap. Fe–TCPP incorporation leads to stronger visible absorption and a red-shifted edge, attributable to porphyrin Soret/Q-band transitions and suggestive of interfacial charge–transfer (CT) absorption across the Fe–N coordination interface. Here, CT refers to the process in which photoexcited electrons in the g-C_3_N_4_ framework are transferred into the low-lying unoccupied orbitals of Fe–TCPP, forming an excited state that spans both components. These CT states are hypothesized based on the optical signatures, and their existence will be further examined in later sections by photoluminescence-based analyses.

From the VB–XPS spectra (Figure 1f), the band edges were obtained using Equations (2) and (3) [41,42]:

(2) $$E_{VB}\left(NHE\right)=\;\psi +E_{VB-XPS}-4.44\;eV$$

(3) $$E_{CB}\left(NHE\right)=E_{VB\left(NHE\right)}-E_{g}$$

With ψ = 4.20 eV, $E_{VB-XPS}\;=\;1.23\;eV\;$(CNNTs) and 1.68 eV (Fe–TCPP), the band positions are: CNNTs: $E_{VB}\;=\;+0.99$, $E_{CB}\;=\;-1.79\;eV$ vs. NHE; Fe–TCPP:$\;E_{VB}\;=\;+1.44$, $E_{CB}=-0.54\;eV$ vs. NHE. These band alignments imply that CNNTs, with a more negative conduction band, could in principle provide sufficient driving force for electron transfer to oxygen species, while the Fe–TCPP conduction band is less favorable for direct O_2_ reduction. By contrast, energy transfer routes that generate ^1O_2_ or secondary processes leading to •OH formation appear more thermodynamically plausible in the composite. At this stage, these are predictions derived from band energetics; their validity will be assessed in later sections through reactive oxygen species (ROS) measurements.

**Figure 2:**
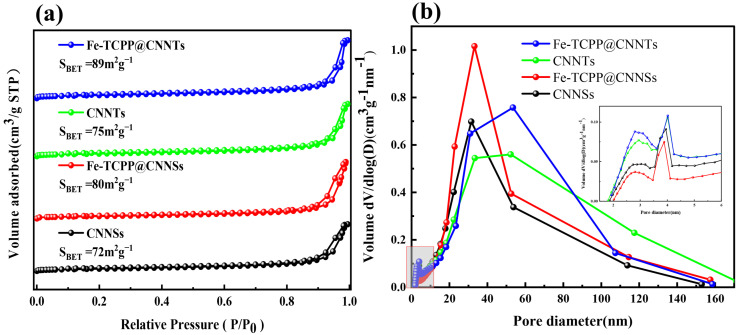
(**a**) N_2_ adsorption–desorption isotherms; (**b**) BJH pore size distribution curves of CNNSs, CNNTs, Fe–TCPP@CNNSs, and Fe–TCPP@CNNTs.

The nitrogen adsorption–desorption isotherms (Figure 2a) of CNNSs, CNNTs, and their Fe–TCPP-modified counterparts exhibit typical type IV behavior with H3-type hysteresis loops, indicative of mesoporous structures derived from the lamellar and tubular frameworks. Compared with the pristine CNNSs (72 m^2^ g^−1^) and CNNTs (75 m^2^ g^−1^), the BET surface areas increase to 80 m^2^ g^−1^ and 89 m^2^ g^−1^ for Fe–TCPP@CNNSs and Fe–TCPP@CNNTs, respectively. This increase can be attributed to the presence of Fe–TCPP molecules, which may help prevent aggregation and contribute to a more open porous structure. The pore size distribution curves (Figure 2b) show that CNNSs and CNNTs have dominant mesopores centered in the 25–35 nm range. After Fe–TCPP modification, the pore size distribution broadens toward 30–60 nm, accompanied by an increase in pore volume. The inset in Figure 2b highlights an increase in micropores and small mesopores (2–10 nm) in the Fe–TCPP composites, which is plausibly related to partial occupation of the framework by porphyrin molecules. Collectively, these structural and optical characterizations confirm that Fe–TCPP incorporation effectively modulates the electronic structure, surface area, and porosity of g-C_3_N_4_, thereby establishing a favorable foundation for enhanced photocatalytic activity.

### 3.2. Morphology Evolution of g-C_3_N_4_

**Figure 3:**
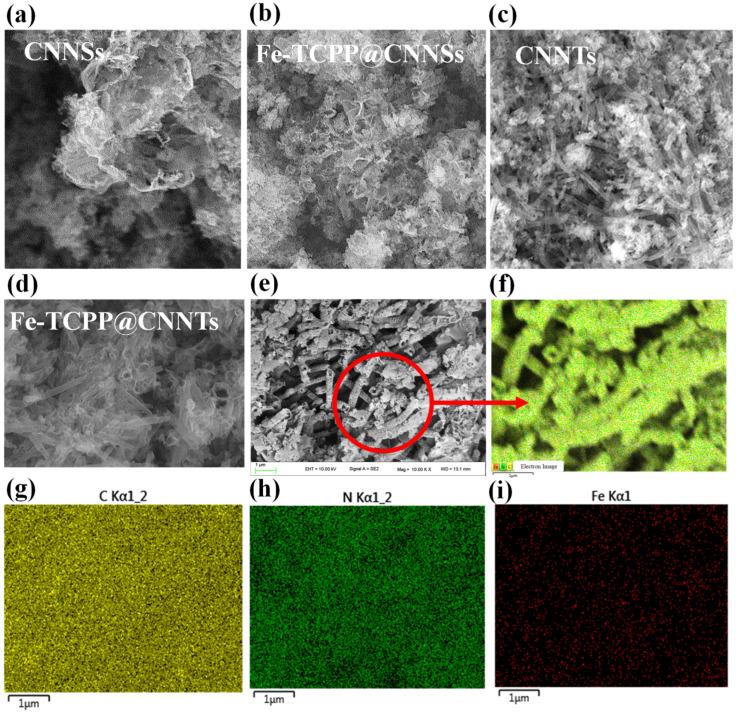
(**a**–**d**) SEM images of (**a**) CNNSs, (**b**) Fe–TCPP@CNNSs, (**c**) CNNTs, and (**d**) Fe–TCPP@CNNTs; (**e**) high-magnification SEM image of Fe–TCPP@CNNTs highlighting surface nanostructures; (**f**–**i**) corresponding EDS elemental mapping images of C, N, and Fe for Fe–TCPP@CNNTs.

The morphology and structural features of CNNSs, CNNTs, and their Fe–TCPP composites were investigated by field-emission scanning electron microscopy (FE-SEM) (Figure 3a–e). CNNSs exhibit thin, wrinkled sheet-like architectures with loosely stacked layers and lateral dimensions up to several hundred nanometers (Figure 3a). After Fe–TCPP modification (Figure 3b), the nanosheets appear roughened and fragmented, suggesting that the porphyrin molecules disturb interlayer stacking and promote surface texturing. By contrast, CNNTs obtained with higher urea content (Figure 3c) display well-defined tubular morphologies with diameters below 100 nm and lengths of several micrometers, consistent with a gas-template-assisted self-rolling process during thermal polycondensation. Fe–TCPP@CNNTs retain this tubular structure (Figure 3d), but their surfaces become rougher and exhibit shallow nanopores, indicating successful surface decoration by Fe–TCPP.

High-magnification SEM images (Figure 3e) reveal fine nanostructured features distributed over the tube surfaces, which can be ascribed to Fe–TCPP domains. This additional roughness increases the density of accessible surface sites, potentially favoring molecular adsorption. Elemental mapping by energy-dispersive X-ray spectroscopy (EDS) (Figure 3f–i) confirms the uniform distribution of C, N, and O, consistent with the g-C_3_N_4_ framework and porphyrin ligands, while Fe signals are also homogeneously dispersed along the nanotubes, indicating effective integration of Fe–TCPP without aggregation or phase separation.

Taken together, these observations confirm that Fe–TCPP molecules are uniformly anchored onto both nanosheet and nanotube g-C_3_N_4_ without inducing structural collapse. Beyond simple geometry, the tubular composites provide a curved two-dimensional framework that introduces curvature-related confinement. Such curvature modifies electronic state distributions compared with flat nanosheets, enhances charge localization, and thereby amplifies quantum-size effects [35,43,44]. These features, in addition to a larger accessible surface area and improved light penetration, make nanotube-based composites a more favorable structural platform. It should also be noted that defects are inevitably present in both CNNSs and CNNTs, and their type and density vary with synthesis conditions. Defects can create localized trap states that accelerate carrier recombination and affect thermal stability, while in some cases they enhance visible-light absorption and provide additional active sites. Recent theoretical modeling confirms that vacancies and structural distortions in carbon nitride frameworks modulate electronic states and stability.

### 3.3. X-Ray Photoelectron Spectroscopy Analysis

**Figure 4:**
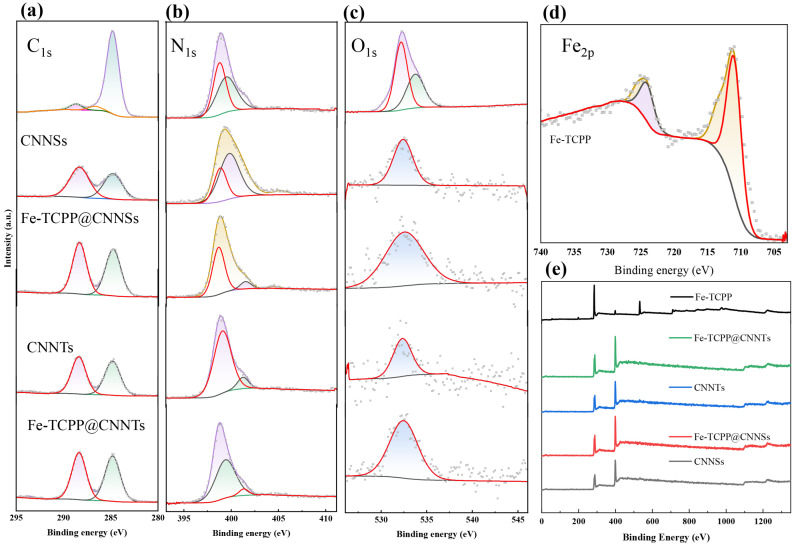
High-resolution XPS spectra of (**a**) C 1s, (**b**) N 1s, (**c**) O 1s, and (**d**) Fe 2p for Fe–TCPP, CNNSs, CNNTs, Fe–TCPP@CNNSs, and Fe–TCPP@CNNTs; (**e**) corresponding survey spectra.

The elemental composition and chemical states were analyzed by XPS. The survey spectrum (Figure 4) confirms the presence of C, N, O, and Fe, with C and N arising from the g-C_3_N_4_ framework and porphyrin ligand, and O and Fe from the metalloporphyrin. To elucidate the interfacial bonding and electronic modulation induced by Fe–TCPP incorporation, high-resolution XPS measurements were performed on Fe–TCPP, CNNSs, CNNTs, and their corresponding composites.

In the C 1s spectra, pristine g-C_3_N_4_ shows two main peaks at ~284.6 eV (adventitious C–C/C=C) and ~288.1 eV, characteristic of sp^2^-hybridized N–C=N linkages. After hybridization with Fe–TCPP, additional features appear at ~285.6 eV (C–N) and ~288.6 eV (C=O), which originate from the porphyrin macrocycle. The enhanced intensity of the C=O peak and the slight shift in the N–C=N component suggests π–π interactions and partial charge delocalization between Fe–TCPP and the g-C_3_N_4_ matrix. The O 1s spectra provide complementary evidence. For pristine CNNSs and CNNTs, a broad signal at ~532.6 eV is assigned to adsorbed water and surface hydroxyl groups. After Fe–TCPP incorporation, the O 1s profile broadens and develops a distinct shoulder at ~531.5 eV, attributable to carbonyl (C=O) groups of the porphyrin. This convolution of hydroxyl- and carbonyl-related signals results in an apparent centroid downshift to ~532.3 eV. The shift is therefore not a sharp single-component displacement, but rather the superposition of multiple oxygen environments arising from interfacial bonding. The N 1s spectra further verify nitrogen-based coordination. CNNSs show three components at 398.3 eV (sp^2^ C=N–C), 399.3 eV (tertiary N–(C)_3_), and 400.6 eV (terminal –NH_x_), together with a minor satellite at 404.6 eV. In Fe–TCPP@CNNSs, the main peak shifts to 398.6 eV, indicating decreased electron density around nitrogen due to Fe–N coordination. The ~400.5 eV peak is significantly suppressed, suggesting reduced hydrogen bonding or amine deprotonation, while the persistence of the satellite peak near 404 eV implies enhanced interfacial excitation or structural disorder. CNNTs, by contrast, show peaks at 398.6, 399.1, and 401.2 eV; after hybridization, these shift to 398.7, 399.5, and 401.4 eV, consistent with similar Fe–N interactions. Notably, the 404 eV satellite is absent in CNNTs and their composites, suggesting a more ordered structure with lower defect density. The Fe 2p spectra of pure Fe–TCPP display two characteristic peaks at ~711.0 eV (Fe 2p_3_/_2_) and ~724.0 eV (Fe 2p_1_/_2_), together with a satellite at ~719.0 eV, all typical of Fe^3+^ species. In the Fe–TCPP@g-C_3_N_4_ composites, however, the Fe 2p signal is not clearly detected, most likely because of the very low Fe loading (1 wt%) combined with the limited surface sensitivity of XPS. Nonetheless, indirect evidence of Fe incorporation is provided by the observed shifts in the N 1s and O 1s regions, which are consistent with Fe–N coordination and porphyrin-related oxygen contributions. These combined spectral features support the successful integration of Fe–TCPP into the g-C_3_N_4_ framework, even though the Fe 2p signal remains below the detection threshold.

Taken together, the coordinated analysis of C 1s, O 1s, and N 1s spectra provides indirect evidence that Fe–TCPP is incorporated into g-C_3_N_4_, with spectral shifts consistent with Fe–N coordination beyond the intrinsic Fe–pyrrole bonding of the porphyrin. In this context, interfacial charge transfer refers to a process in which photoexcited electrons in g-C_3_N_4_ may migrate into the low-lying orbitals of Fe–TCPP through Fe–N coordination, creating a new state spanning both components. The subtle differences between nanosheet- and nanotube-based composites suggest that morphology modulates the interfacial electronic structure, which is expected to influence charge separation and photocatalytic behavior.

### 3.4. Photocatalytic Activity, Stability, and Reproducibility

**Figure 5:**
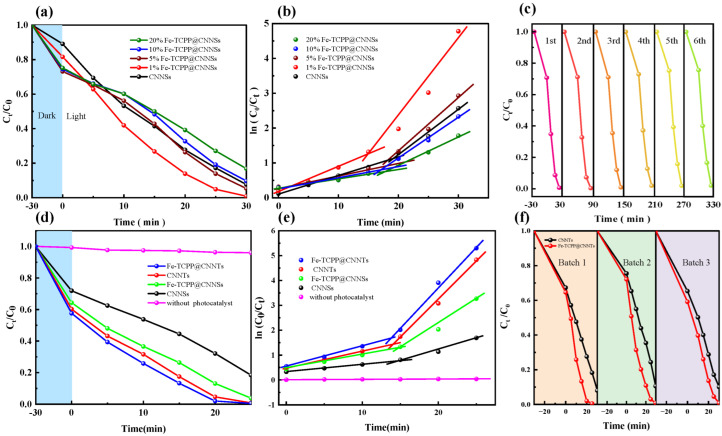
Photocatalytic degradation performance of Fe–TCPP@g-C_3_N_4_ composites under visible-light irradiation: (**a**) Degradation curves of RhB for CNNSs with different Fe–TCPP loadings (1–20 wt%); (**b**) Corresponding two-stage pseudo-first-order kinetic fitting; (**c**) Cycling stability of Fe–TCPP@CNNTs over six consecutive runs; (**d**) Comparison of degradation curves for CNNSs, CNNTs, and their Fe–TCPP-modified counterparts (1 wt%); (**e**) Corresponding kinetic fitting and apparent rate constants; (**f**) Reproducibility test of Fe–TCPP@CNNTs prepared from three independent batches.

To determine the optimal Fe–TCPP loading, a series of Fe–TCPP@CNNSs composites with 1–20 wt% Fe–TCPP were synthesized and evaluated for Rhodamine B (RhB) degradation under visible-light irradiation (Figure 5a,b and Table S1). Compared with pristine CNNSs, all Fe–TCPP-modified samples exhibited enhanced dark adsorption, with the maximum adsorption efficiency reaching ~27% for the 20 wt% composite. However, the photocatalytic activity followed a volcano-type trend with increasing Fe–TCPP content. The 1 wt% Fe–TCPP@CNNSs exhibited the best performance, achieving 99% removal within 30 min, whereas higher loadings (≥5 wt%) resulted in progressively reduced efficiency, most likely due to Fe–TCPP aggregation, surface coverage, or competition for visible-light absorption.

To gain further insight into the reaction kinetics, the degradation processes were analyzed using a pseudo-first-order kinetic model, as described by Equation (4):

(4) $$\ln\left(\frac{c_{0}}{c_{t}}\right)\;=\;kt$$

where $C_{0}$ and $C_{t}$ are the initial and residual concentrations of RhB at time t, and k is the apparent rate constant. The kinetic fitting results clearly support the photocatalytic trend: the 1 wt% Fe–TCPP@CNNSs sample exhibited the highest kinetic constants of *k*_1_ = 0.0749 min^−1^ (initial stage) and *k*_2_ = 0.229 min^−1^ (later stage). In contrast, the 20 wt% sample showed significantly lower values (*k*_1_ = 0.0263 min^−1^, *k*_2_ = 0.0727 min^−1^). These results confirm that while moderate Fe–TCPP loading improves charge utilization, excessive incorporation suppresses charge transfer and compromises reaction kinetics.

Building on the optimal 1 wt% loading, the influence of g-C_3_N_4_ morphology was further assessed by comparing nanosheet- and nanotube-based systems (Figure 5d,e). The Fe–TCPP@CNNTs composite achieved complete RhB degradation within 30 min under visible light, outperforming Fe–TCPP@CNNSs and all other controls. Kinetic analysis gave the highest values among all tested samples (*k*_1_ = 0.0964 min^−1^, *k*_2_ = 0.328 min^−1^), demonstrating that the tubular framework significantly enhances adsorption, light harvesting, and directional charge transport. These findings highlight a strong synergistic effect between Fe–N coordination and nanotubular morphology. The durability of Fe–TCPP@CNNTs was then evaluated by six consecutive RhB degradation cycles (Figure 5c). The catalyst retained over 90% of its initial activity after the sixth run, with only negligible variation in kinetic constants. Post-reaction SEM images (Figure S2) confirmed that the nanotube morphology was preserved without aggregation or collapse, while FT-IR spectra (Figure S3) showed unchanged vibrational signatures of both the g-C_3_N_4_ framework and Fe–TCPP moieties. These results indicate that both the morphology and chemical structure of the composite remain stable during repeated use. Although the Fe content in the catalyst is extremely low (~1 wt% in 20 mg), making direct detection of possible leaching by XPS or ICP difficult, the consistent photocatalytic performance and preserved structural features strongly suggest that no significant Fe loss occurred under the tested conditions. Finally, reproducibility was validated by comparing three independently synthesized batches of Fe–TCPP@CNNTs (Figure 5f). All batches exhibited nearly identical RhB degradation efficiencies and kinetic constants, confirming the robustness of the self-assembly method and the reliability of the photocatalytic performance.

In summary, both Fe–TCPP loading and g-C_3_N_4_ morphology play decisive roles in governing photocatalytic activity. An optimal Fe–TCPP content of 1 wt% yields the best balance between surface reactivity and light absorption, while the nanotubular architecture amplifies these effects through curvature-related confinement and improved charge transport. The reproducible activity across batches and stable performance over multiple cycles provide solid experimental evidence that Fe–TCPP@CNNTs are a reliable and durable platform for visible-light photocatalysis.

### 3.5. Carriers Generation Transfer and Recombination

**Figure 6:**
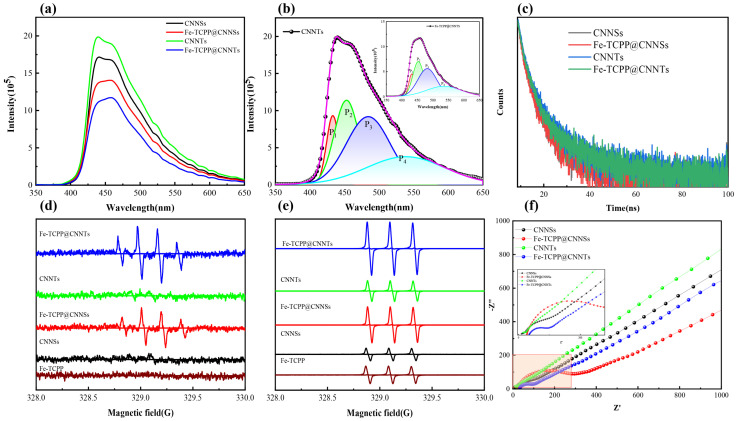
(**a**) Steady-state PL spectra; (**b**) Gaussian-fitted PL spectra; (**c**) Time-resolved photoluminescence (TRPL) decay curves; (**d**,**e**) ESR spectra of CNNSs, CNNTs, and their Fe–TCPP composites under visible-light irradiation using DMPO as a spin-trapping agent, confirming •OH and ^1^O_2_ generation; (**f**) Nyquist plots from electrochemical impedance spectroscopy (EIS) indicating interfacial charge transfer resistance.

The photoluminescence (PL) spectra of CNNSs, CNNTs, and their Fe–TCPP composites exhibit distinct emission characteristics that correlate with morphology and coordination effects (Figure 6a,b and Table S3). Both CNNSs and CNNTs display four Gaussian-resolved emission peaks centered at approximately 433, 454, 485, and 539 nm, while after Fe–TCPP incorporation these peaks shift slightly to 432, 453, 482, and 535 nm, reflecting local electronic perturbations induced by Fe–N coordination. In addition to these peak shifts, the relative contributions of the emission components are altered: the π–π* transition peak at ~433 nm (P1) decreases from 11.1% to 9.2% in CNNSs and from 10.7% to 9.6% in CNNTs, whereas the long-wavelength peak at ~538 nm (P4), commonly assigned to defect-related or ligand-to-metal charge–transfer (LMCT) states, increases from 25.9% to 27.0% in CNNSs and remains high (25.9%) in Fe–TCPP@CNNTs. These spectral evolutions, together with overall PL quenching, suggest that Fe–N coordination introduces additional non-radiative decay channels and stabilizes intermediate trap states, thereby suppressing direct radiative recombination. The effect is more pronounced in Fe–TCPP@CNNTs, which can be attributed to the curved tubular framework facilitating stronger electronic coupling between g-C_3_N_4_ and Fe–TCPP. Taken together, these features are consistent with the formation of interfacial charge–transfer states, where photogenerated electrons are more effectively separated from holes and partially stabilized in defect- or LMCT-related levels, providing a rational explanation for the enhanced charge separation efficiency and laying the foundation for the improved photocatalytic performance observed in subsequent sections.

Time-resolved photoluminescence (TRPL) measurements (Table S4 and Figure 6c) further corroborate the role of morphology and coordination in charge–carrier dynamics. Transformation from nanosheets (CNNSs) to one-dimensional nanotubes (CNNTs), followed by Fe–TCPP incorporation, systematically prolongs both the fast and slow decay components: τ_1_ increases from 2.12 ns (CNNSs) to 2.40 ns (CNNTs) and 2.61 ns (Fe–TCPP@CNNTs), while τ_2_ extends from 9.90 ns to 10.42 ns and 12.55 ns, respectively. These prolonged lifetimes indicate suppressed non-radiative recombination and more efficient separation of photogenerated carriers, in line with earlier reports on metalloporphyrin-modified g-C_3_N_4_ systems. Mechanistically, the nanotubular framework provides anisotropic pathways that facilitate directional charge transport and reduce recombination at structural defects, whereas Fe–N coordination introduces intermediate states that serve as charge–transfer channels, delaying carrier recombination and promoting migration toward surface redox sites. The synergistic effect of these two factors thus explains the superior photocatalytic activity of Fe–TCPP@CNNTs, where prolonged lifetimes translate directly into enhanced charge utilization during visible-light-driven reactions.

Electron paramagnetic resonance (EPR) spectroscopy with spin-trapping agents was employed to identify reactive oxygen species (ROS) generated under visible-light irradiation (Figure 6d,e). Using DMPO in an aqueous solution, a characteristic quartet signal with a 1:2:2:1 intensity ratio was observed, indicative of hydroxyl radicals (•OH); the signal intensity increased significantly in Fe–TCPP@g-C_3_N_4_ composites, particularly in the nanotube form, whereas Fe–TCPP alone showed no response. Given that the valence band potentials of g-C_3_N_4_ (<+1.5 V vs. NHE) are insufficient for direct •OH generation from water oxidation, the detected •OH is attributed to secondary routes, including the conversion of DMPO–OOH (formed by initial trapping of superoxide, •O_2_^−^) into DMPO–•OH during irradiation, as well as surface-mediated reactions of •O_2_^−^/HO_2_• with adsorbed water or hydroxyl groups at defect sites [45,46]. Complementary TEMP spin-trapping experiments further confirmed the production of singlet oxygen (^1^O_2_), as evidenced by the characteristic 1:1:1 triplet pattern, with Fe–TCPP@CNNTs showing the strongest signal due to efficient Type-II energy transfer from Fe–TCPP* to O_2_ and morphology-enhanced charge/oxygen transport. Collectively, these results establish that •O_2_^−^ and ^1^O_2_ are the dominant ROS under visible-light irradiation, while •OH is generated via secondary pathways yet still contributes to the overall photocatalytic activity.

Electrochemical impedance spectroscopy (EIS, Figure 6f) was employed to probe the charge–-transfer properties of the composites. In the high-frequency region, only Fe–TCPP-modified samples exhibit distinct semicircles, characteristic of interfacial charge–transfer resistance (Rct). Among them, Fe–TCPP@CNNTs presents the smallest semicircle, indicating reduced Rct and more efficient charge separation due to the combined effects of tubular morphology and Fe–N coordination, whereas Fe–TCPP@CNNSs shows comparatively larger resistance. Pristine CNNSs display only slight curvature, while CNNTs approach a vertical line, reflecting transport-dominated behavior with negligible interfacial resistance. In the low-frequency region, all samples exhibit Warburg-type diffusion features, with Fe–TCPP@CNNTs showing the steepest slope, consistent with the fastest ionic diffusion and charge transport. The nearly identical high-frequency intercepts on the Z′ axis confirm that electrolyte resistance (Rs) remains similar across all samples. Overall, the EIS results demonstrate that coupling Fe–TCPP with g-C_3_N_4_ nanotubes substantially lowers charge–transfer resistance and accelerates interfacial carrier migration, providing a mechanistic basis for the superior photocatalytic performance of Fe–TCPP@CNNTs.

## 4. Conclusions

**Scheme 2:**
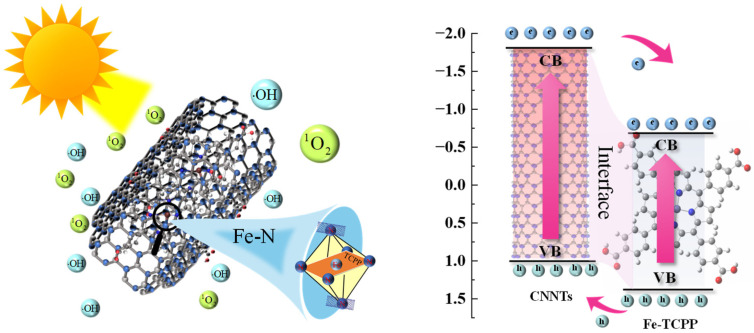
Schematic illustration of photocatalytic ROS generation over Fe–TCPP@CNNTs.

The incorporation of Fe–TCPP into g-C_3_N_4_ nanotubes (CNNTs) markedly enhances photocatalytic efficiency through the synergistic effects of morphology and coordination. The optimized 1 wt% Fe–TCPP@CNNTs achieved the highest apparent rate constants, several times higher than unmodified CNNTs, supported by structural and surface analyses showing larger specific surface area, uniform mesoporosity, and anisotropic charge-transport channels. Optical and electronic characterizations confirmed extended visible-light absorption, favorable band alignment, prolonged carrier lifetimes, and reduced charge–transfer resistance, providing a consistent picture of enhanced charge separation. EPR and scavenger experiments further identified superoxide radicals (•O_2_^−^) and singlet oxygen (^1^O_2_) as the dominant reactive oxygen species, with •OH generated mainly via secondary pathways, highlighting the crucial role of interfacial charge–transfer states mediated by Fe–N coordination and tubular confinement. These results are summarized in a schematic illustration of photocatalytic ROS generation over Fe–TCPP@CNNTs (Scheme 2). These combined structural and electronic advantages establish a robust mechanistic basis for the superior photocatalytic performance of Fe–TCPP@CNNTs and demonstrate a broadly applicable strategy for designing high-efficiency photocatalysts for environmental remediation, solar energy conversion, and selective oxidative transformations.

## Data Availability

The data supporting the findings of this study are available within the article and its Supplementary Materials. Additional raw data are available from the corresponding author upon reasonable request.

## Supplementary Materials

The following supporting information can be downloaded at: https://www.mdpi.com/article/10.3390/nano15191465/s1, Figure S1: Tauc plots for estimating the optical band gaps of CNNSs, Fe–TCPP@CNNSs, CNNTs, Fe–TCPP@CNNTs, and Fe–TCPP, derived from UV–vis diffuse reflectance spectra; Figure S2: FE-SEM images of Fe–TCPP@CNNTs recorded (a) before cycling; (b) after six photocatalytic cycles. Figure S3: FT-IR spectra of Fe–TCPP@CNNTs (a) before cycling; (b) after six photocatalytic cycles. Figure S4: Gaussian deconvolution of the steady-state photoluminescence (PL) spectra for (a) CNNSs, (b) Fe–TCPP@CNNSs, (c) CNNTs, and (d) Fe–TCPP@CNNTs. All spectra were resolved into four emission components (P_1_–P_4_), corresponding to different radiative recombination pathways. The changes in peak position and relative intensity reflect the influence of Fe–TCPP coordination and morphological effects on charge carrier recombination dynamics. Table S1: Dark adsorption efficiencies, photocatalytic degradation efficiencies, and pseudo-first-order rate constants (k_1_, k_2_) of Fe–TCPP@CNNSs with varying Fe–TCPP loadings (1–20 wt%) under visible-light irradiation; Table S2: Dark adsorption efficiencies, photocatalytic degradation efficiencies, and pseudo-first-order rate constants (k_1_, k_2_) of CNNSs, CNNTs, their Fe–TCPP composites, and the control sample without catalyst; Table S3: Peak positions and area ratios of Gaussian-fitted PL spectra for CNNSs, CNNTs, and their Fe–TCPP composites, indicating emission transition shifts and intensity changes induced by Fe–N coordination and morphology; Table S4: Lifetimes (τ_1_, τ_2_) and relative contributions of photogenerated carriers for CNNSs, CNNTs, and their Fe–TCPP composites, obtained from TRPL measurements.

## Abbreviations

The following abbreviations are used in this manuscript:

CNNSs	g-C_3_N_4_ nanosheets
CNNTs	g-C_3_N_4_ nanotubes
ROS	Reactive oxygen species
PL	Photoluminescence
TRPL	Time-resolved photoluminescence
EPR	Electron paramagnetic resonance
BET	Brunauer–Emmett–Teller
XRD	X-ray diffraction
FTIR	Fourier-transform infrared spectroscopy
XPS	X-ray photoelectron spectroscopy
