# Supplementary material for: Morphology–Coordination Coupling of Fe–TCPP and g-C3N4 Nanotubes for Enhanced ROS Generation and Visible-Light Photocatalysis
Source: Nanomaterials (Basel). 2025 Sep 24;15(19):1465. doi: 10.3390/nano15191465 (PMC12525746; doi:10.3390/nano15191465)
Supplement: Supplementary file 1 [file nanomaterials-15-01465-s001.zip › nanomaterials-3847572-supplementary.pdf]

Supplementary Information

Morphology–Coordination Coupling of Fe–TCPP and g-C_3N_4 Nanotubes for Enhanced ROS Generation and Visible-Light Photocatalysis

Nannan Zheng [1], Yulan Zhang [1], Chunlei Dong [1], Zhiming Chen [2], Jianbin Chen[1,*]

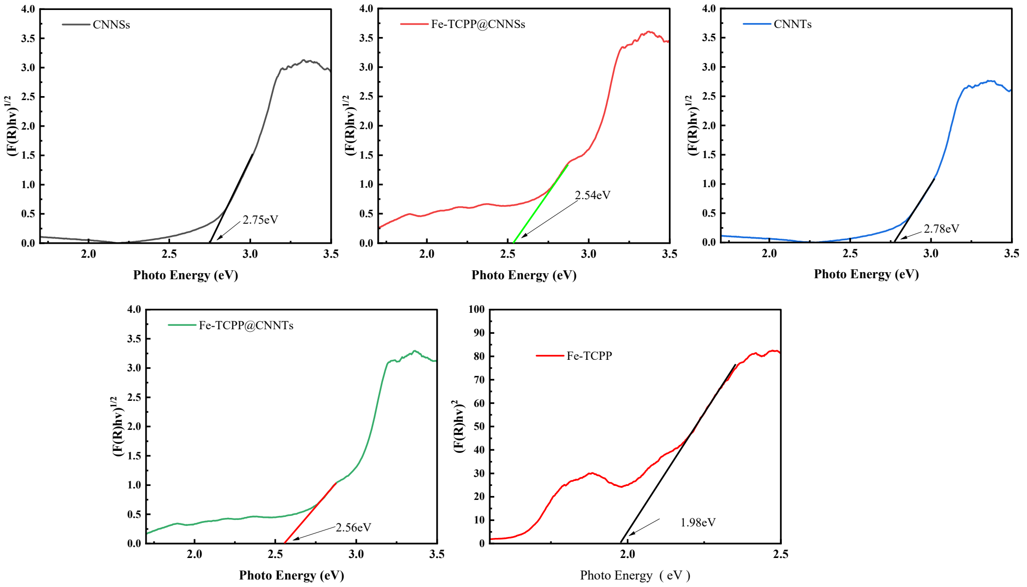

Figure S1. Tauc plots for estimating the optical band gaps of CNNSs, Fe-TCPP@CNNSs, CNNTs, Fe-TCPP@CNNTs, and Fe-TCPP, derived from UV–vis diffuse reflectance spectra.

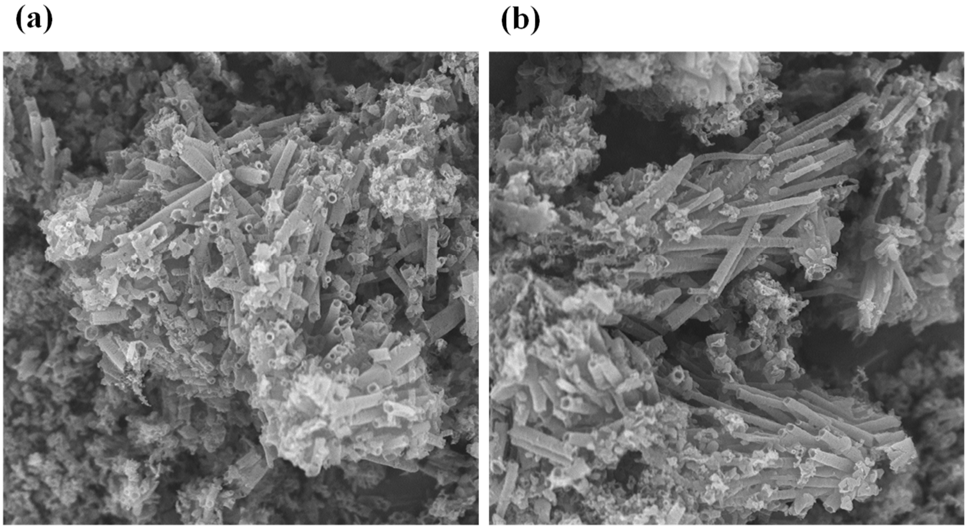

Figure S2. FE-SEM images of Fe-TCPP@CNNTs recorded(a) before cycling; (b) after six photocatalytic cycles.

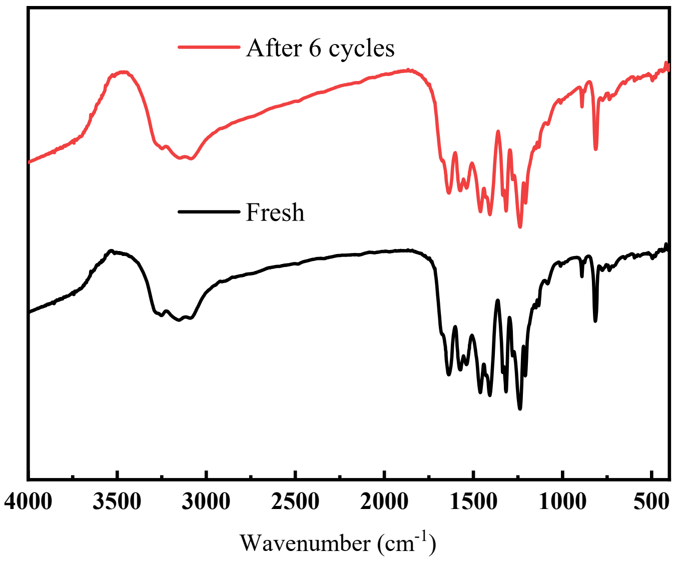

Figure S3. FT-IR spectra of Fe-TCPP@CNNTs (a) before cycling; (b)after six photocatalytic cycles.

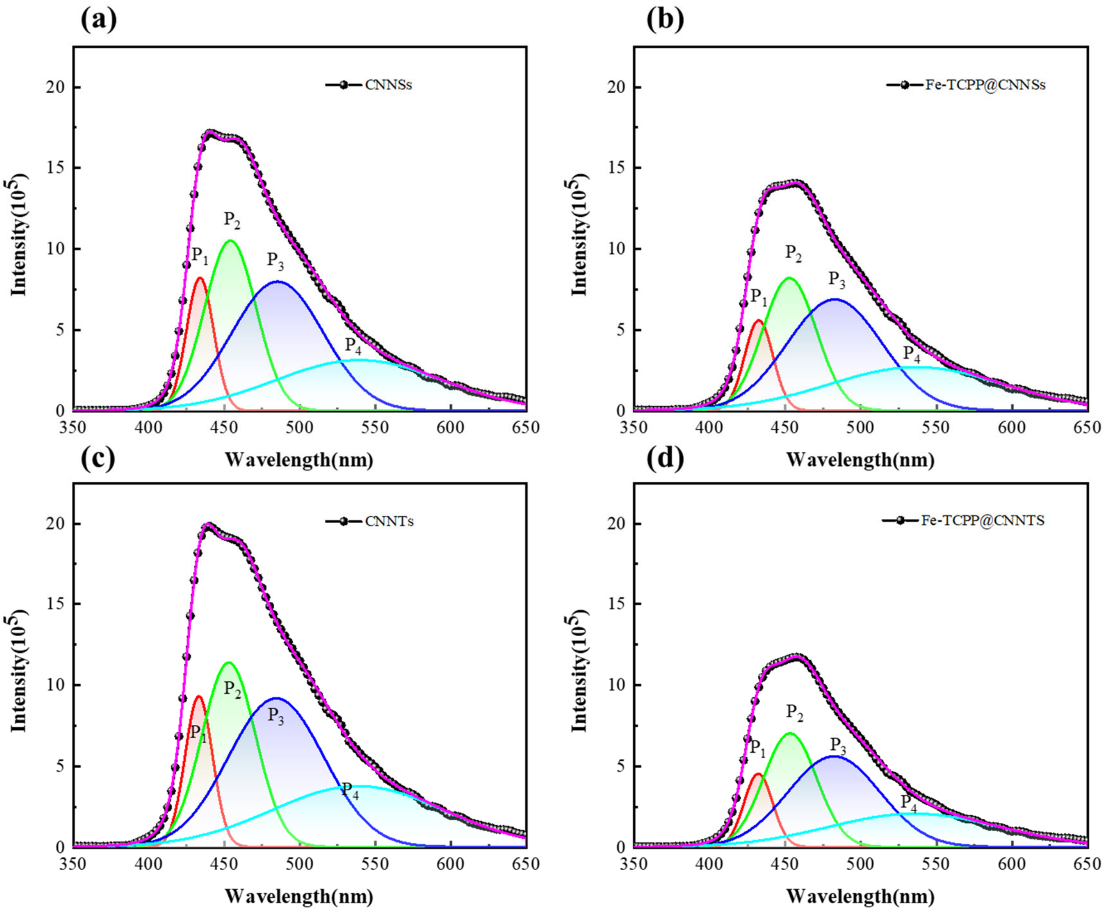

Figure S4. Gaussian deconvolution of the steady-state photoluminescence (PL) spectra for a) CNNSs, b) Fe-TCPP@CNNSs, c) CNNTs, and d) Fe-TCPP@CNNTs. All spectra were resolved into four emission components (P_1–P_4), corresponding to different radiative recombination

pathways. The changes in peak position and relative intensity reflect the influence of Fe-TCPP coordination and morphological effects on charge carrier recombination dynamics.

Table S1 Dark adsorption efficiency, photocatalytic degradation efficiency, and pseudo-first-order rate constants (k_1, k_2) of Fe-TCPP@CNNSs with varying Fe-TCPP loadings (1–20 wt%) under visible-light irradiation.

Sample	Dark adsorption efficiency (%)	Photocatalytic degradation efficiency (%)	Initial Stage k_1 (min^{-1})	Later Stage k_2 (min^{-1})
CNNSs	11	92	0.0512	0.111
**1% Fe-TCPP@CNNSs**	18	99	0.0749	0.229
5% Fe-TCPP@CNNSs	27	95	0.0352	0.138
10% Fe-TCPP@CNNSs	26	90	0.0278	0.107
20% Fe-TCPP@CNNSs	25	83	0.0263	0.0727

Table S2 Dark adsorption efficiency, photocatalytic degradation efficiency, and pseudo-first-order rate constants (k_1, k_2) of

Sample	Dark adsorption efficiency (%)	Photocatalytic degradation efficiency (%)	Initial Stage k_1 (min^{-1})	Later Stage k_2 (min^{-1})
Without catalyst	0.7	4	0.00132	0.00124
**CNNSs**	28	82	0.0318	0.0881
Fe-TCPP@CNNSs	36	96	0.0589	0.194
CNNTs	40	99	**0.0805**	**0.309**
Fe-TCPP@CNNTs	43	100	**0.0964**	**0.328**

Table S3 Peak positions and corresponding area ratios of Gaussian-fitted photoluminescence (PL) spectra for CNNSs, CNNTs, and their Fe-TCPP composites. Peaks P_1–P_4 represent different emission transitions, with shifts in wavelength and intensity ratios reflecting variations in electronic structure and recombination behavior induced by Fe–N coordination and morphological differences.

**Peak**	**CNNSs (nm)**	**Area Ratio (%)**	**Fe-TCPP @CNNSs (nm)**	**Area Ratio (%)**	**CNNTs (nm)**	**Area Ratio (%)**	**Fe-TCPP @CNNTs (nm)**	**Area Ratio (%)**
**P1**	433.93	11.1	432.46	9.17	433.11	10.7	432.16	9.55
**P2**	454.02	26.9	452.78	25.5	452.95	25.6	453.15	27.6
**P3**	485.28	36.2	482.65	37.7	484.32	36.7	482.20	38.0
**P4**	539.24	25.9	536.68	27.0	538.45	27.0	535.34	25.9

Table S4 Lifetimes (τ_1, τ_2) of photogenerated charge carriers for CNNSs, CNNTs, and their Fe-TCPP composites as derived from time-resolved photoluminescence (TRPL) measurements.

**Samples**	**Component**	**Life Time (ns)**	**Relative percentage（%）**	χ^2
**CNNSs**	τ_1	2.12	55.33	1.2145
	τ_2	9.90	44.67	
**Fe-TCPP@CNNSs**	τ_1	2.24	55.93	1.238
	τ_2	10.72	44.07	
**CNNTs**	τ_1	2.40	49.53	1.0950
	τ_2	10.42	50.47	
**Fe-TCPP@ CNNTs**	τ_1	2.61	57.17	1.1907
	τ_2	12.55	42.83	
